# Pneumatospinning and Electrospinning Scaffolds for Meniscus Regeneration Using Human Embryonic-Derived Mesenchymal Stem Cells

**DOI:** 10.3390/bioengineering13030314

**Published:** 2026-03-09

**Authors:** Shawn P. Grogan, Erik W. Dorthé, Austin B. Williams, Nicholas E. Glembotski, Darryl D. D’Lima

**Affiliations:** Scripps Health, Shiley Center for Orthopaedic Research and Education at Scripps Clinic, 10666 North Torrey Pines Road, La Jolla, CA 92037, USA; grogan.shawn@scrippshealth.org (S.P.G.); dorthe.erik@scrippshealth.org (E.W.D.); auwilliams@scripps.edu (A.B.W.); glembotski.nicholas@scrippshealth.org (N.E.G.)

**Keywords:** meniscus regeneration, embryonic stem cell-derived mesenchymal stem cells, tissue engineering, collagen scaffolds, pneumatospun scaffolds, heparin, growth factor immobilization, ex vivo, pushout mechanical testing

## Abstract

We evaluated human embryonic stem cell-derived mesenchymal stem cells (ES-MSCs) on collagen scaffolds for meniscus-like neotissue formation and ex vivo repair of human osteoarthritic (OA) meniscal defects. Collagen type I fibrous scaffolds were pneumatospun, and laminate scaffolds were fabricated from electrospun PLA/collagen; crosslinked; heparin conjugated; fibronectin coated; functionalized with TGFβ1, TGFβ3, or PDGFbb; seeded with ES-MSCs; and cultured for 4 weeks, followed by in vitro assessment or ex vivo implantation into 3.5 mm human meniscus defects for 5 weeks. Pneumatospinning generated highly porous scaffolds that supported uniform cell infiltration, while laminate scaffolds demonstrated interlocking fiber interfaces and enhanced mechanical properties. TGFβ1 and TGFβ3 immobilization enhanced scaffold bioactivity, defined as growth factor-mediated increases in meniscus-like matrix deposition, collagen fiber organization, and meniscogenic gene expression, by significantly increasing safranin O staining, collagen type II deposition, collagen fiber polarization, and ACAN expression. TGFβ3 additionally increased COL1A1 expression and pushout shear modulus; TGFβ1 increased peak pushout stress, indicating superior ex vivo mechanical integration. Laminate scaffolds resulted in extensive cell infiltration, robust neotissue formation (elastic modulus ~2.4 MPa), and improved ex vivo tissue integration when functionalized with TGFβ3. The data indicated that ES-MSC-seeded, heparin-conjugated, TGFβ-immobilized pneumatospun/electrospun collagen–PLA scaffolds support meniscogenic differentiation and biomechanical integration, with repair of focal meniscal defects and potential for partial meniscus replacement.

## 1. Introduction

The meniscus is a critical fibrocartilaginous structure in the knee joint, responsible for load distribution, shock absorption, and joint stability [1]. Injury or degeneration of menisci often leads to osteoarthritis (OA) [2]. Meniscal tears are among the most common knee injuries. Recent data from the USA estimates the incidence at approximately 61 per 100,000 in the general population [3,4]. Clinical evidence indicates that repair of meniscal tears is recommended over meniscectomy [5,6], with improved outcomes observed in the vascular regions of the menisci [7,8]. However, repair of avascular tears remains challenging [9,10].

Cell-based therapies combined with various scaffolds have emerged as promising tools for meniscus regeneration, especially using mesenchymal stem cells derived from knee joint tissues [11,12,13]. We and others have explored nanofibrous scaffolds in combination with a variety of cell sources for evidence of neotissue regeneration. Most of these scaffolds are produced by electrospinning a variety of natural and synthetically derived biomaterials to fabricate tissue-engineered meniscus scaffolds (see review [14]). Synthetic polymers include polycaprolactone (PCL), polyurethane (PU), polylactic acid (PLA), polyglycolic acid (PGA), and polylactic-co-glycolic acid (PLGA). Naturally sourced materials include meniscus extracellular matrix (ECM) and polymers such as silk fibroin (SF), chitosan (CS), collagen, and hyaluronic acid (HA), with the latter two being major constituents of natural meniscus. A combination of natural and synthetic materials has also been used to produce a template for cellular attachment and tissue regeneration along with sufficient mechanical properties to mimic natural meniscus tissue (see reviews [15,16]).

Nanofibrous electrospun scaffolds are attractive in meniscus tissue engineering because they replicate key aspects of the native extracellular matrix, such as the biochemical composition and ultrastructural organization. However, they present significant limitations: (i) their dense nanometer-scale fiber networks hinder cellular infiltration and (ii) constrain scaffold thickness, which typically does not exceed 1 mm, thereby limiting their applicability in full-thickness meniscus repair [17,18]. To address these issues, strategies such as incorporating sacrificial fibers, porosity-inducing additives, or multilayer assembly have been investigated. While these approaches can enhance cellular infiltration, they often compromise mechanical integrity or necessitate complex fabrication techniques [19,20,21].

To overcome some of the inherent issues using electrospun scaffolds, we and others have explored pneumatospinning, which uses air pressure to generate collagen fibers [22,23]. We demonstrated that pneumatospinning can rapidly produce scaffolds (1 mm thick within 2 h) with a porosity that enabled excellent cell seeding and distribution. Human adipose-derived stem cells seeded on pneumatospun nanofibrous collagen scaffolds produced meniscus-like neotissues that integrated into bovine and human ex vivo meniscal explants [23].

A variety of cell types have been investigated for meniscus tissue engineering, including meniscus-derived cells and various sources of mesenchymal stem cells (MSCs). We have previously explored human meniscus cells [15,24] and MSCs from a variety of tissue sources, including adipose (infrapatellar fat), bone marrow, and synovium, as candidate populations for meniscus regeneration [23,25]. Other groups have reported the use of IPFP-MSC [26], synovium-derived MSCs [27,28], and peripheral blood-derived MSCs [29], all of which demonstrate considerable potential for enhancing meniscus repair. Despite their promise, these cell sources exhibit several practical and clinical limitations, such as donor site morbidity [30], restricted expansion and proliferation capacity [31,32], and potential for undesired (off-target) differentiation [33]. As an alternative, we have developed a xenofree embryonic stem cell line that can be differentiated to an MSC phenotype exhibiting multilineage capacity, including chondrogenic and mineralized cartilage differentiation [34]. These cells provide a renewable and potentially universal source for meniscus tissue engineering.

To enhance tissue regeneration, several methods have been used to incorporate bioactive molecules such as growth factors (GFs) into scaffold matrices. Numerous immobilization techniques have been developed, including physical adsorption, covalent conjugation, and layer-by-layer assembly [35,36,37], which were often combined with heparin affinity methods to optimize release kinetics and biological effect. We have previously shown that heparin conjugation enhances meniscus tissue regeneration [38,39].

We and others have evaluated the potential of various scaffolds and cells for generating neotissue and repairing tissue defects in animal and human ex vivo meniscus explants [15,23,39,40]. While exploring cellular responses, these models have also examined the relationship between cellular responses and integration with host tissues using mechanical pushout testing, primarily with porcine meniscus [40,41,42,43,44,45] and with human meniscus tissues [46].

The principal aim was to combine a pneumatospun collagen scaffold with clinically relevant embryonic-derived MSCs (ES-MSCs) and to enhance bioactivity by immobilizing growth factors using heparin conjugation. We evaluated neotissue formation and mechanical integration to analyze the effect of growth factor immobilization in a human OA meniscus explant model. A secondary aim was to test proof of concept of fabricating a laminate scaffold that would enhance mechanical properties while satisfying biocompatibility, cell colonization, and neotissue formation, with potential for use in partial meniscus replacements.

## 2. Methods

### 2.1. Pneumatospinning Spinning Setup and Collagen Scaffold Fabrication

**Figure 1:**
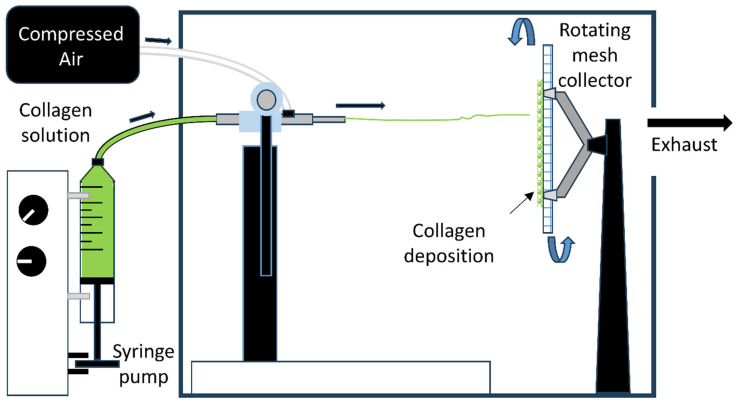
Overview of pneumatospinning device. Schematic of the system for pneumatospinning collagen fibrous scaffolds. A box enclosure controls humidity and temperature and exhausts the solvent fumes. Collagen solution is delivered via a syringe pump to the central channel of the coaxial nozzle. Compressed air is delivered into the outer channel of the coaxial nozzle to deposit collagen fibers onto the stainless-steel mesh collector to form a fibrous mat over time (12–24 h).

An overview of the pneumatospinning device is shown in Figure 1 and Supplemental Data Figure S1. The experimental setup was adapted from Dorthé et al., 2022 [23], for better control of solution feed rates. A coaxial needle was assembled such that the inner, 28-gauge needle was connected to a syringe pump filled with the working solution, while the outer, 14-gauge needle was connected to compressed air regulated to 30 psi. The tip of the inner needle extended 10 mm beyond the tip of the outer needle. Collagen type I (Semed S, generously supplied by DSM Biomedical, Exton, PA, USA) was dissolved in hexafluoroisopropanol (HFIP, Sigma Aldrich, St. Louis, MO, USA) at a concentration of 9% (*w*/*v*), as previously optimized for spinning [23]. This solution was loaded into a 50 mL syringe connected to the inner needle and fed at a rate of 8 µL/min. The target was a square section of flat stainless-steel mesh with a 0.012” wire diameter (TWP Inc., Berkeley, CA, USA) mounted on an electric motor and rotated at 650 RPM, with the axis of rotation colinear with the axis of the nozzle. The distance between the nozzle and target was set to 12 cm (Figure 1). Pneumatospun collagen scaffolds were crosslinked with glutaraldehyde (GA, 25%, Thermo Fisher Scientific, Waltham, MA, USA) vapor by suspending the scaffold over an open beaker containing 30 mL GA solution warmed to 40 °C. This assembly was enclosed in a glass container for 48 h to contain the GA vapor and kept in a chemical fume hood. After GA crosslinking, scaffolds were cut to size, washed four to five times in Hanks’ Balanced Salt Solution (HBSS, Thermo Fisher Scientific, Waltham, MA, USA), and stored in HBSS at 4 °C for subsequent experiments.

### 2.2. Coaxial Electrospinning

Coaxial scaffolds were electrospun in a SpinBox (Nanoscience Instruments, Phoenix, AZ, USA) (Supplemental Data Figure S1). Coaxial scaffolds were composed of polylactic acid (PLA, Ingeo 4043D, NatureWorks, Plymouth, MN, USA) and collagen type I (Semed S, DSM Biomedical), as detailed previously [15]. Briefly, 10% (*w*/*v*) PLA in HFIP and 7% (*w*/*v*) collagen (Semed S, DSM Biomedical, Exton, PA, USA) in HFIP were used as the core and shell solutions, respectively, through the Fluidnatek Coaxial Nozzle (Fluidnatek, Valencia, Spain). The environmental conditions were 54% relative humidity and 19.8 °C. The tip of the needle was 11 cm from the rotating drum collector (diameter = 100 mm). The positive voltage was set to 11 KV, the negative voltage was set to −0.5 KV, the drum was set to rotate at 1000 RPM, the core solution was extruded at 1 mL/h, and the shell solution was extruded at 0.3 mL/h. Scaffolds were removed from the drum collector and stored in a sealed plastic bag at 4 °C.

### 2.3. Laminate Scaffold Fabrication

**Figure 2:**
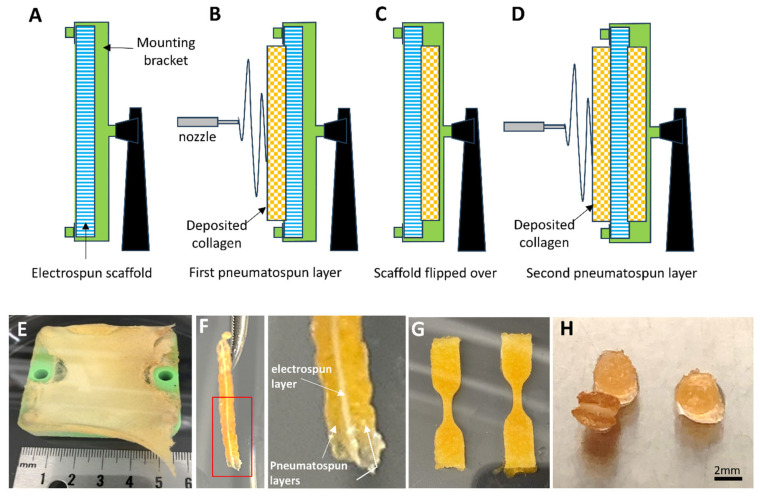
Laminate scaffold fabrication. (**A**) An electrospun coaxial scaffold (PLA and collagen) is mounted on a custom frame. (**B**) A layer of collagen fibers is pneumatospun on one surface of the electrospun scaffold. (**C**) The two-layered scaffold is flipped over on the frame. (**D**) Another layer of collagen fibers is pneumatospun to produce a tri-layered laminate scaffold. (**E**) The laminate scaffolds are crosslinked using glutaraldehyde fumes for 48 h. (**F**) Photograph of laminate scaffold after hydration. In set shows the coaxial scaffold between pneumatospun collagen layers. (**G**) Laminate scaffolds cut as “dog-bone” shapes for mechanical testing. (**H**) Laminate scaffold cut into 3.5 mm diameter by 2–3 mm thick discs for ES-MSC seeding and ex vivo human meniscus implantation.

**Figure 3:**
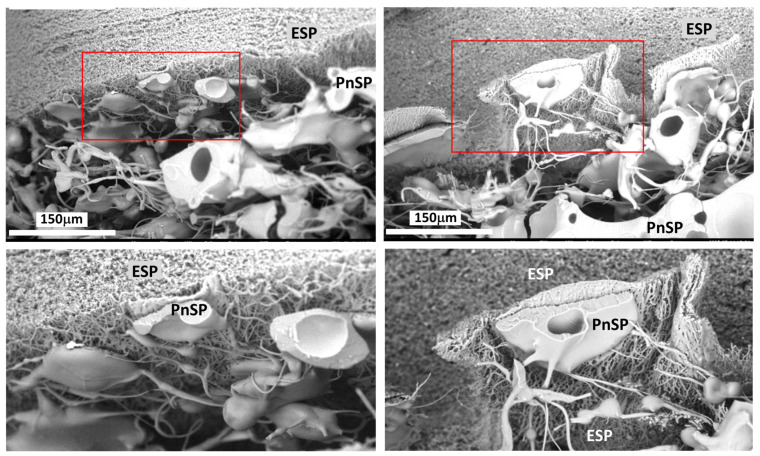
Scanning Electron Microscopy (SEM) of laminate scaffold interface. **Top row:** Representative SEM images of the laminate scaffold interface between the upper electrospun (ESP) and lower pneumatospun scaffold (PnSP). Original magnification = 1000×. **Bottom row:** Higher magnification of interface region (depicted by red rectangles in the top row) showing interdigitation of pneumatospun fibers with coaxial electrospun fibers.

Laminate scaffolds were produced using the same method as the pneumatospun collagen scaffolds, with the following modifications. Coaxial electrospun scaffolds (PLA and collagen) were cut and mounted in place of the stainless-steel mesh using a custom 3D-printed clamping frame (Figure 2). A layer of pneumatospun collagen (9%) was deposited onto the coaxial scaffold until 25 mL of collagen solution had been dispensed for approximately 12 h. The scaffold was then inverted for deposition of the remaining 25 mL onto the other side of the coaxial scaffold. The newly formed laminate scaffold was crosslinked using GA vapor as described above. The ultrastructure of the interface between pneumatospun and electrospun scaffolds was examined under scanning electron microscopy (SEM) (Figure 3).

### 2.4. Heparinization and Fibronectin Coating of Scaffolds

The GA crosslinked pneumatospun collagen scaffolds and the laminate scaffolds were heparinized as outlined previously [39,47]. Collagen scaffolds (maximum 10 mm in diameter) were equilibrated with 0.05 M 2-morpholinoethane sulfonic acid buffer (MES, pH 5.6, Sigma-Aldrich, Burlington, MA, USA) for 30 min. Heparin sodium salt was dissolved at a concentration of 3 mg/mL in 0.05 M MES buffer containing 25 mM EDC/10 mM NHS to activate the carboxyl groups of heparin. Depending on scaffold size, at least three times the volume of the heparin solution was added to each sample, and the samples were incubated overnight at room temperature with gentle shaking to allow covalent conjugation between the carboxyl groups of heparin and the amine groups of collagen. The scaffolds were rinsed three times with 0.1 M Na_2_HPO_4_, pH 7.0 in distilled water. The heparinized scaffolds were washed three times with HBSS before soaking the scaffold in HBSS with 10 mg/mL human fibronectin (from human plasma; Sigma-Aldrich) overnight at 4 °C or until ready for use.

### 2.5. Cell Source

Human embryonic-derived mesenchymal stem cells (ES-MSCs) were differentiated from an ESC cell line as previously reported [34]. Briefly, xenofree derived ESC [48] (HADC-100 ESC) were cultured as cell clusters for 5 days with an ALK-5 inhibitor (10 mM; SB525334; Selleckchem, Houston, TX, USA) and then cultured in 2D on fibronectin-coated flasks in serum-free medium (Stem Pro-34, Thermo Fisher Scientific, Carlsbad, CA, USA) supplemented with bFGF (20 ng/mL; Thermo Fisher, Carlsbad, CA, USA). The cells that emerged from the clusters displayed a typical MSC surface marker profile (CD73, CD90, and CD105 positive; CD34 and CD45 negative) and displayed chondrogenic and osteogenic differentiation capacity. The ES-MSCs in this study were used between passage 3 and 5.

### 2.6. Growth Factor Immobilization and Scaffold Cell Seeding

**Figure 4:**
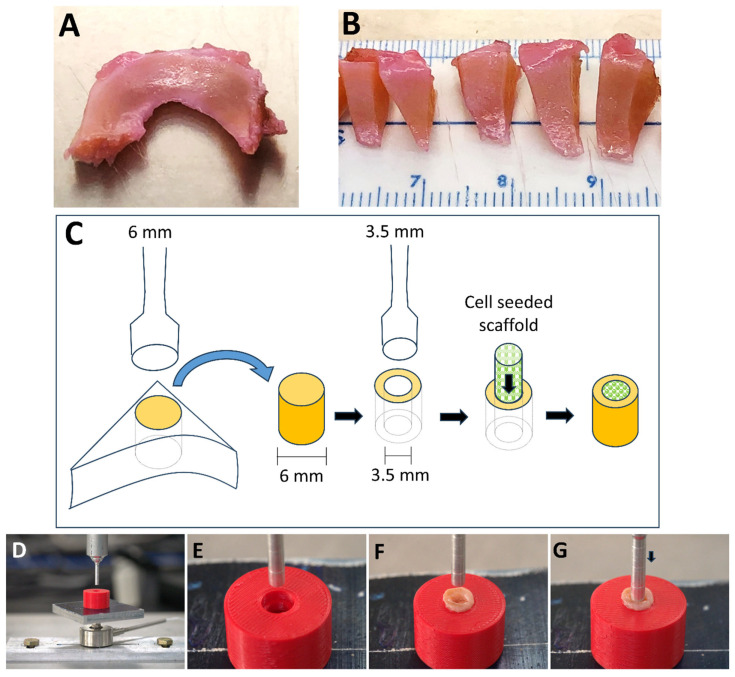
Ex vivo human osteoarthritic (OA) meniscus model. (**A**,**B**) Human OA meniscus tissues were harvested for ex vivo culture. (**C**) Dermal punches were used to obtain hollow cylindrical explants with an outer diameter of 6 mm and inner diameter of 3.5 mm. Cylindrical scaffolds (3.5 mm in diameter × 2–3 mm thickness) seeded with ES-MSCs were implanted in the center of the hollow explants and cultured for 5 weeks. (**D**–**G**) Photographs of the mechanical pushout testing. A custom holder (**E**) was used to mount the ex vivo implanted tissue (**F**). A cylindrical rod (3 mm) pushed out the implanted scaffolds (**G**) while measuring the force and displacement of the rod.

Pneumatospun scaffolds that were glutaraldehyde crosslinked, heparinized, and fibronectin coated were cut into 3.5 mm discs (2–3 mm thick) and soaked in one of the following growth factors: TGFβ1, TGFβ3, or PDGFbb (Peprotech, Rocky Hill, NJ, USA) at 100 ng/mL in HBSS for 12 h. Control scaffolds were only heparinized and fibronectin coated. Scaffolds were washed in HBSS prior to cell seeding. ES-MSCs were seeded onto scaffolds (N = 4) at a density of 0.5 × 10^6^ per well in multiple wells of a 12-well ultra-low non-adherent (ULAP) plate (Corning Incorporated, Kennebunk, ME, USA) and placed on an orbital shaker (Ohaus Parsippany, NJ, USA) at 150 RPM for 7 days in ES-MSC expansion medium to permit cell infiltration into the scaffolds and proliferation. Thereafter, the scaffolds were placed into differentiation medium consisting of Dulbecco’s Modified Eagle Medium (DMEM) (Mediatech Inc., Manassas, VA, USA), 1 × ITS + 1 supplement (Sigma-Aldrich), 100 nM dexamethasone (Sigma-Aldrich), 100 µM ascorbic acid 2-phosphate (Sigma-Aldrich), 1.25 mg/mL human serum albumin (Bayer, Leverkusen, Germany), and 1% Penicillin–Streptomycin–Gentamycin (Life Technologies, Carlsbad, CA, USA) and then supplemented with 10 ng/mL TGFβ3 (Peprotech, Thermo Fisher Scientific, Cranbury, NJ, USA) and cultured for a minimum of 14 days before being implanted into 3.5 mm defects made in human OA meniscus tissue (Figure 4). These were all cultured for 5 weeks in the differentiation medium, with medium changes every 3–4 days.

Laminate scaffolds were similarly glutaraldehyde crosslinked, heparinized, fibronectin coated, and soaked in TGFβ3 at 100 ng/mL in HBSS. The laminate scaffolds were prepared either for ex vivo implantation (3.5 mm diameter × 2–3 mm thick disc) or for free culture (10 mm long, 0.5 mm wide, and 3–4 mm thick). The cell seeding approach was the same as described for the pneumatospun collagen scaffolds. To facilitate uniform cell distribution in the larger free-cultured scaffolds, cells were seeded onto both sides of the scaffold over several days. After 3 days of an initial seeding of 1 × 10^6^ cells in a 12-well ULAP, the scaffold was turned over, and another 1 × 10^6^ ES-MSCs were added. Laminate scaffolds were cultured in expansion medium for 7–10 days on an orbital shaker and then transferred to differentiation medium for 14 days before implantation into meniscus defects or maintained in free culture for 5 weeks.

### 2.7. Human Meniscus Ex Vivo Tissues

To simulate an in vivo repair environment, human osteoarthritic menisci were obtained from patients following total knee arthroplasty within 4–6 h after surgery (approved by Scripps Health Institutional Review Board). For this study, a total of 16 donors were utilized (N = 8 female, average age 70.6 ± 7.9 years; and N = 8 male, average age 73.8 ± 7.0 years). Fresh menisci were cut into sagittal sections (4–6 mm thick), and cylindrical explants were harvested with 6 mm dermal punches. An inner concentric defect was created in each explant with a 3.5 mm dermal punch (see Figure 4). The explants were maintained in culture medium consisting of DMEM (Mediatech Inc., Manassas, VA, USA) supplemented with 10% calf serum (Omega Scientific Inc., Tarzana, CA, USA) and 1% Penicillin–Streptomycin–Gentamycin (Life Technologies, Carlsbad, CA, USA) prior to implantation. Pneumatospun collagen scaffold cylinders (3.5 mm diameter and 2–3 mm thick) containing ES-MSCs were implanted into the central defect of each implant and cultured in serum-free medium with TGFβ3 (10 ng/mL) for 5 weeks, with medium changes every 3–4 days.

### 2.8. Mechanical Testing Pushout Model

Following ex vivo culture for 5 weeks, the integration of the implanted cell-seeded scaffolds was assessed using a pushout mechanical test (Figure 4) [40]. Meniscus explant and scaffold constructs were placed in a holder that supported the 6 mm diameter explant ring. A 3 mm cylindrical punch was then lowered onto the scaffold, which was pushed out at 0.18 mm/min while forces were recorded using a uniaxial mechanical tester (Biomomentum, Laval, QC, Canada). Shear modulus was calculated during pushout, and peak stress was recorded when the force dropped below 10% of peak force.

### 2.9. Mechanical Testing of Laminate Scaffolds

Laminate scaffolds were cut into dog-bone-shaped specimens (Figure 2G) using a custom-made aluminum template as described previously [24]. Briefly, dog-bone specimens were gripped in a uniaxial mechanical tester (Biomomentum, Laval, QC, Canada) and subjected to tensile loading until rupture. Elastic modulus and peak stress before failure were calculated.

### 2.10. Viability

To assess the cell attachment and overall cytotoxicity to the scaffolds, a live–dead assay was performed. Calcein-AM and Ethidium Homodimer-1 (Live/Dead kit, Life Technologies, Carlsbad, CA, USA) were used with the scaffold tissues and imaged using a fluorescent microscope (Axiovert 200 M, Zeiss, Oberkochen, Germany). The percentage of live and dead cells was calculated using ImageJ/(version 1.54P; National Institutes of Health, Bethesda, MD, USA) after image processing by thresholding and segmentation as described previously [23].

### 2.11. Mouse In Vivo Subcutaneous Implantation

All animal experiments were performed in compliance with protocols approved by the institutional animal care and use committee at the Scripps Research Institute. Pneumatospun scaffolds, seeded with ES-MSCs (N = 6) or acellular scaffolds (N = 6), were implanted subcutaneously in the dorsum of nude mice through skin incisions. Corresponding constructs without cells served as controls. Implants were harvested 6 weeks after implantation. The quality of the neotissue was assessed by histologic stains (Safranin O for glycosaminoglycans) and IHC (for collagen types I and II).

### 2.12. Histology and Immunohistochemistry

Scaffolds and ex vivo tissue explants were fixed in Z-Fix (3.7% formaldehyde, Anatech Ltd., Battle Creek, MI, USA) for 24–48 h, processed for embedding in paraffin, and cut into 4 μm sections. Sections were stained with Safranin O and Fast Green to visualize glycosaminoglycan distribution in the tissues. Immunostaining for detection of collagen types I and II was performed as described previously [34]. Sections were pretreated with pepsin (Digest-All 3, Thermo Fisher Scientific, Carlsbad, CA, USA) for 9 min at 37 °C in a humid chamber before incubation at 4 °C for 12 to 16 h with the following primary antibodies: rabbit anti-human collagen type I antibody (Ab 34710, Abcam, Cambridge, MA, USA) 1 µg/mL or mouse anti-human collagen type II (II-II6B3, Hybridoma Bank, University of Iowa, Iowa City, IA, USA) 2 µg/mL. For color development, the ImmPRESS secondary DAB kit (Vector Laboratories, Burlingame, CA, USA) was used. Isotype controls were used to monitor non-specific staining.

### 2.13. Histomorphometry

Images of the ex vivo Safranin O stained or immunostained for collagen type I or type II were quantified using ImageJ (version 1.54P, National Institutes of Health, Bethesda, MD, USA). For Safranin O, the color channels were separated using color deconvolution, and the red channel was selected. The polygon tool was used to manually outline the interface between the host and implanted scaffolds, restricting the region of interest to the implant. Background thresholding was applied to separate the implant from empty space, and percent positive Safranin O signal was recorded.

To quantify the immunostained sections for collagens type I and type II, the above process was repeated with the following modifications. DAB brown deconvolution was used, and the brown channel was selected. Results were normalized against the isotype controls from adjacent histological sections.

### 2.14. Histology Grading

Neotissue formation and integration was graded using the Ishida grading system [49] with the following criteria (total grade ranging from 0 to 6):

(1) Reparative tissue bonding: No bonding = 0 points; Partial bonding with surrounding meniscus = 1 point; Bilateral bonds with surrounding meniscus = 2 points.
(2) Existence of fibrochondrocytes: No fibrochondrocytes in reparative tissue = 0 points; Fibrochondrocytes are localized in the reparative tissues = 1 point; Fibrochondrocytes exist diffusely in the reparative tissues = 2 points.
(3) Staining with Safranin O: Not stained with Safranin O = 0 points; Faintly stained with Safranin O = 1 point; Densely stained with Safranin O = 1 points.

### 2.15. RNA Extractions and Gene Expression

RNA extractions were performed by homogenizing the cell-seeded scaffolds using the Qiashredder and the RNAeasy kit (Qiagen Valencia, CA, USA). Complementary DNA (cDNA) was synthesized using a High Capacity cDNA Reverse Transcription Kit (Applied Biosystems, Foster City, CA, USA). Gene expression of COL1A1, COL2A1, COMP, ACAN, CHAD, THY-1, and MKX was measured using pre-validated TaqMan^®^ gene expression reagents (Applied Biosystems, Westminster, CO, USA) using the Lightcycler 96 real time PCR device (Roche, Basel, Switzerland). The expression levels were normalized to GAPDH using the method as previously reported [50], and the relative change in gene expression in the neotissues was compared to the expression levels of undifferentiated ES-MSCs in the monolayer culture.

### 2.16. Polarized Light Analysis

Picrosirius red stained sections (N = 6–8) were analyzed using polarized light microscopy to assess collagen fiber organization and alignment. Sections were imaged using a Nikon Optiphot microscope (Nikon Corporation, Tokyo, Japan) equipped with crossed linear polarizers, with digital images captured using an AmScope MU2003-BI camera (AmScope, Irvine, CA, USA) at 10× magnification. Two regions of interest (ROIs) were acquired for each section: scaffold-only regions (S) and the interface between the scaffold and meniscus tissue explant (Int). ROIs were selected to ensure complete image coverage following rotational analysis.

Collagen fiber alignment was analyzed as previously reported [51]. For each ROI, a series of seven images were acquired at polarizer angles of 0°, 15°, 30°, 60°, 90°, 120°, 150°, and 180° relative to a fixed analyzer orientation. Light intensity measurements were recorded for each angular position, yielding maximum intensity I_max_ and minimum intensity I_min_ values for each ROI. The degree of polarization (DoP) was calculated using the following formula:

$$DoP=\frac{I_{max}-I_{min}}{I_{max}+I_{min}}$$

where DoP values range from 0 (random fiber orientation) to 1 (perfect fiber alignment).

### 2.17. Scanning Electron Microscopy (SEM)

Pneumatospun and laminate scaffolds were fixed in 2.5% glutaraldehyde solution in PBS for 18 h and preserved in 70% ethanol at 4 °C. For SEM, the scaffolds were dehydrated in 100% ethanol, twice for 30 min, then in a 1:1 ratio of 100% ethanol and hexamethyldisilazane (HMDS, Sigma Aldrich, Burlington, MA, USA) for 30 min, followed by two incubations in 100% HMDS for 30 min each. Before imaging, the scaffold was placed in a multi-well plate in a fresh volume of 100% HMDS and dried overnight (~14–16 h). Dehydrated scaffolds were mounted on aluminum posts using conductive adhesive and imaged using SEM (Phenom Pro, Thermo Fisher Scientific, Waltham, MA, USA).

SEM images were analyzed as previously described [23]. Briefly, pore sizes were measured across the longest dimension using ImageJ software (version 1.54P; National Institutes of Health, Bethesda, MD, USA). Pore sizes within each image were averaged to obtain a single representative value. A threshold value was applied to distinguish foreground (scaffold material) from background (pore space) regions of the image, with background sections considered regions of porosity. Percent porosity was quantified by measuring the background area fraction across four linear profiles per image, and values were averaged to yield a single porosity measurement for each image.

### 2.18. Statistical Analysis

The statistical significance of differences between gene expression, histomorphometric analysis, histology, polarized light, and mechanical measurements was calculated using ANOVA and paired *t*-tests. *p*-values less than 0.05 were considered significant.

## 3. Results

### 3.1. Pneumatospun and Electrospun Scaffolds Produced Unique Fiber Geometries with Biocompatible Properties

**Figure 5:**
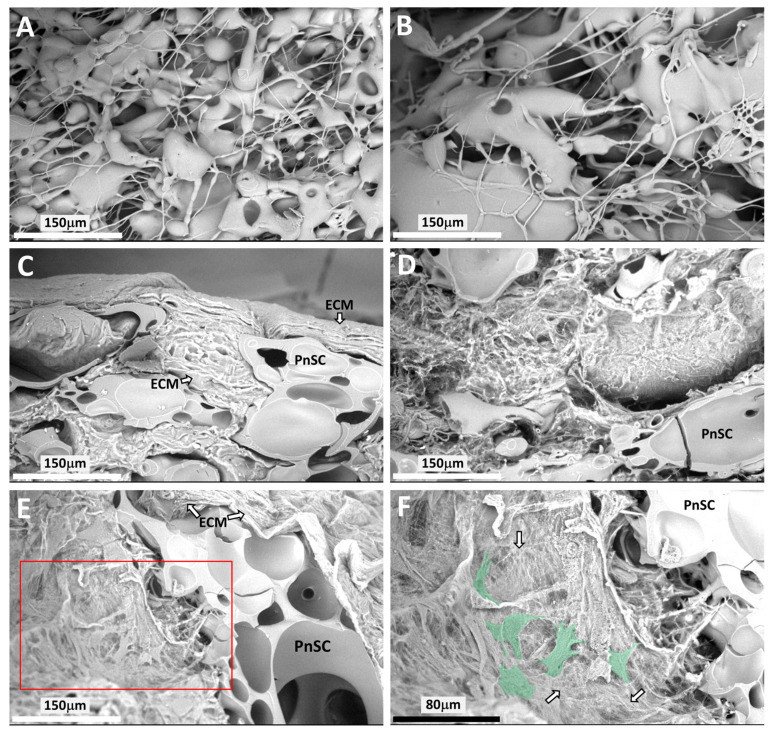
Scanning electron microscopy (SEM) of pneumatospun collagen scaffolds (PnSC). (**A**,**B**) Image showing the porosity of scaffold without cells. (**C**–**E**) ES-MSCs seeded into pneumatospun scaffolds generated neotissues that filled the pores after 5 weeks of culture (magnification 1000×; white scale bar 150 μm). (**F**) Higher magnification of panel (**E**) (red box) showing cells (green) and fibrous extracellular matrix (white arrows) (magnification 1850×; black scale bar 80 μm).

SEM of acellular scaffolds revealed characteristics similar to those we had reported previously [23]. Pneumatospun fibers appeared irregular with a wide range of fiber sizes and fibers that changed diameter over their length (Figure 5A,B). The overall porosity of the collagen scaffold in the transverse plane was approximately 28%, with a median pore size of 24.4 μm and a mean of 26.5 μm. SEM of ES-MSC-seeded pneumatospun collagen scaffolds after 5 weeks showed cells with fibroblast-like morphology and extensive ECM production within and on the surface of the collagen scaffold (Figure 5C–F).

**Figure 6:**
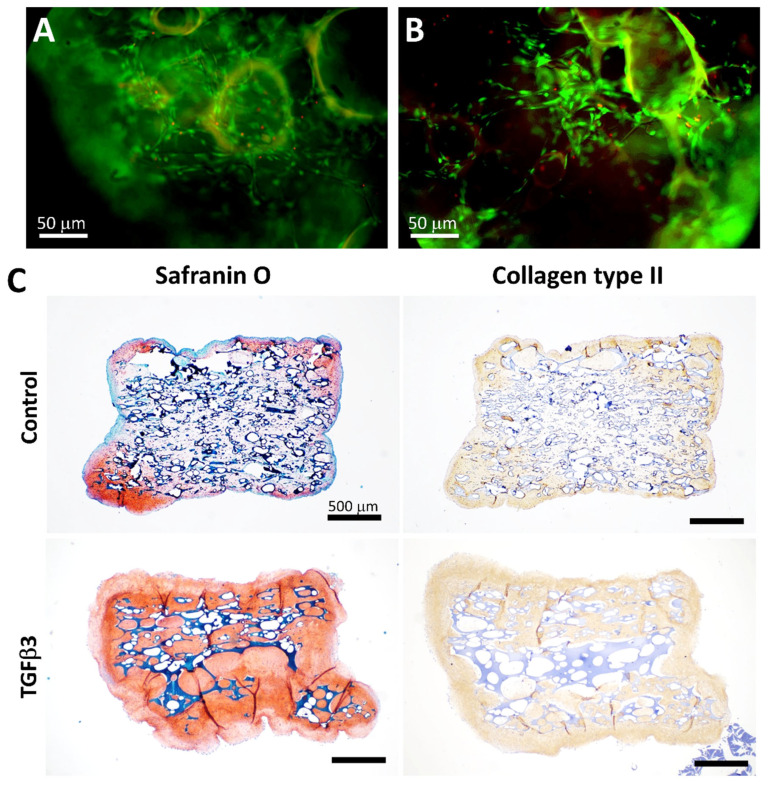
Establishing conditions for cell attachment and robust neotissue formation on pneumatospun collagen scaffolds using ES-MSCs. (**A**,**B**) Scaffolds (3.5 mm × 2–3 mm thick discs) were coated with fibronectin and show high cell viability (85 ± 5%) (white scale bar = 50 mm). (**C**) ES-MSCs were seeded upon the scaffolds for a total of 5 weeks. For the first week, cells were permitted to infiltrate and proliferate into the scaffold. For the last 4 weeks, the scaffolds and cells were cultured in differentiation medium. Uniform cell infiltration and deposition of neotissues after 5 weeks in culture with Safranin O positive stain (red-orange stain) and collagen type II ECM deposition (brown stain) (black scale bar = 500 µm). More robust neotissue formation was observed in heparinized scaffolds conjugated (conj.) with TGFβ3.

The ES-MSCs are cultivated upon fibronectin in 2D cultures [34]. To enhance cell attachment, we coated the scaffolds (3.5 mm wide; 2–3 mm thick) under the same conditions (10 mg/mL). We previously observed that heparin conjugation of growth factors enhanced cellular activities [39]; hence, we heparin conjugated the scaffolds to immobilize TGFβ3 on the scaffolds. Fibronectin and heparin treatments enhanced cell attachment and distribution throughout the scaffold over 5 weeks, resulting in neotissue formation with high cell viability (Figure 6). Although fibronectin coating alone was sufficient to promote positive cell activities, TGFβ3 immobilization clearly enhanced neotissue formation, with more extensive Safranin O and collagen type II deposition (Figure 6).

### 3.2. Growth Factor Immobilization Enhances Ex Vivo Neotissue Regeneration

**Figure 7:**
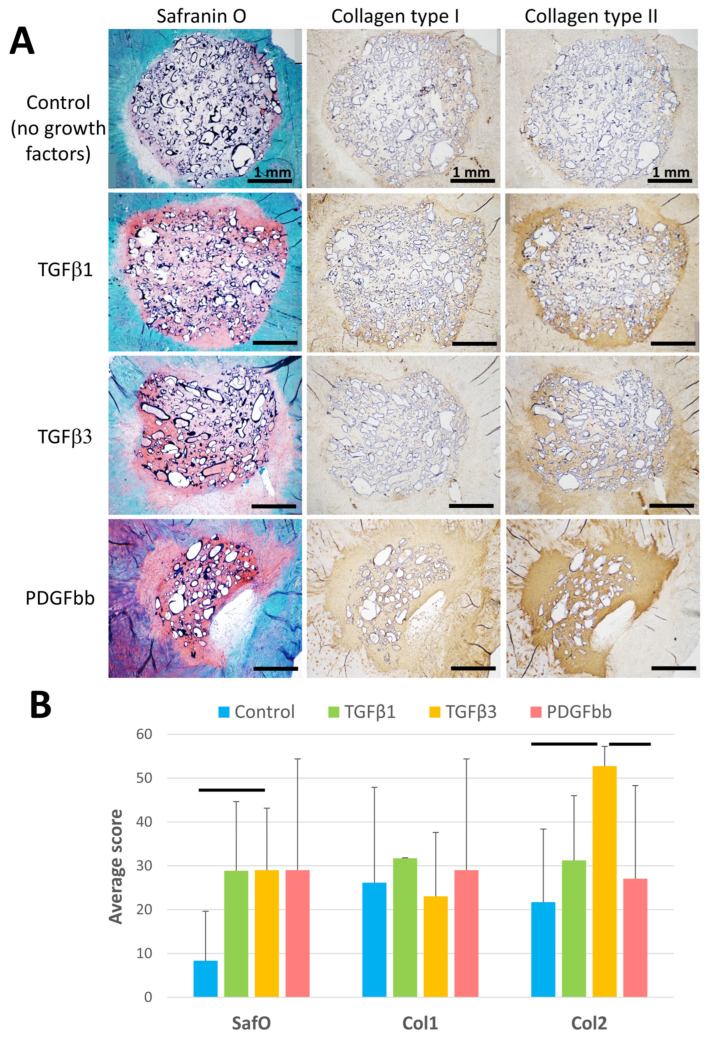
Histology and histomorphometric analysis of ex vivo meniscus tissue repair with heparinized pneumatospun collagen scaffolds. Scaffolds treated with different growth factors and seeded with ES-MSCs were implanted ex vivo into human meniscus tissue defects for 5 weeks. (**A**) Sections stained with Safranin O (red-orange positive stain) and immunostained for collagen types I and II (brown positive stain). Scale bar = 1 mm. (**B**) Histomorphometric image analysis scores (mean ± SD). The horizonal bars indicate significant differences between the 2 groups at the ends of each bar.

Growth factor-immobilized scaffolds (TGFβ1, TGFβ3 or PDGFbb) were implanted into 3.5 mm defects in human OA meniscus explants (see Figure 4). After 5 weeks of ex vivo culture, the histology of the explants (N = 5–6 for each condition) showed the formation of neotissues, with all growth factor treatments resulting in positive staining for Safranin O and both collagen types I and II (Figure 7A) relative to controls (without growth factors, Figure 7A). Histomorphometric measurements (Figure 7B) indicated significantly greater Safranin O staining in scaffolds with TGFβ (*p* < 0.02), while that in scaffolds with PDGFbb approached significance (*p* < 0.08). Ishida grading resulted in a significantly greater score for all growth factor treatments in comparison to controls (*p* < 0.01; control grades 4.5 ± 0.6; all others 6.0 ± 0.0). No significant difference in staining intensity was observed for collagen type I staining between all treatments (*p* > 0.2). A significant increase in collagen type II was found in scaffolds containing TGFβ3 (*p* < 0.04) and PDGFbb (*p* < 0.03).

The degree of polarization (DoP) was calculated within the scaffold and at the interface between the scaffold and the native meniscus tissue. In comparison to controls without growth factors, a significantly higher DoP was observed for TGFβ treatments (TGFβ1 *p* < 0.005; TGFβ3 *p* < 0.02). TGFβ treatments also resulted in significantly higher DoP values compared to the PDGFbb treatment (TGFβ1 *p* < 0.004; TGFβ3 *p* < 0.02). DoP at the scaffold–meniscus interface was not significantly different between growth factor-treated groups, although TGFβ3 treatment approached significance for higher DoPs (*p* < 0.07, Supplemental Data Figure S2).

**Figure 8:**
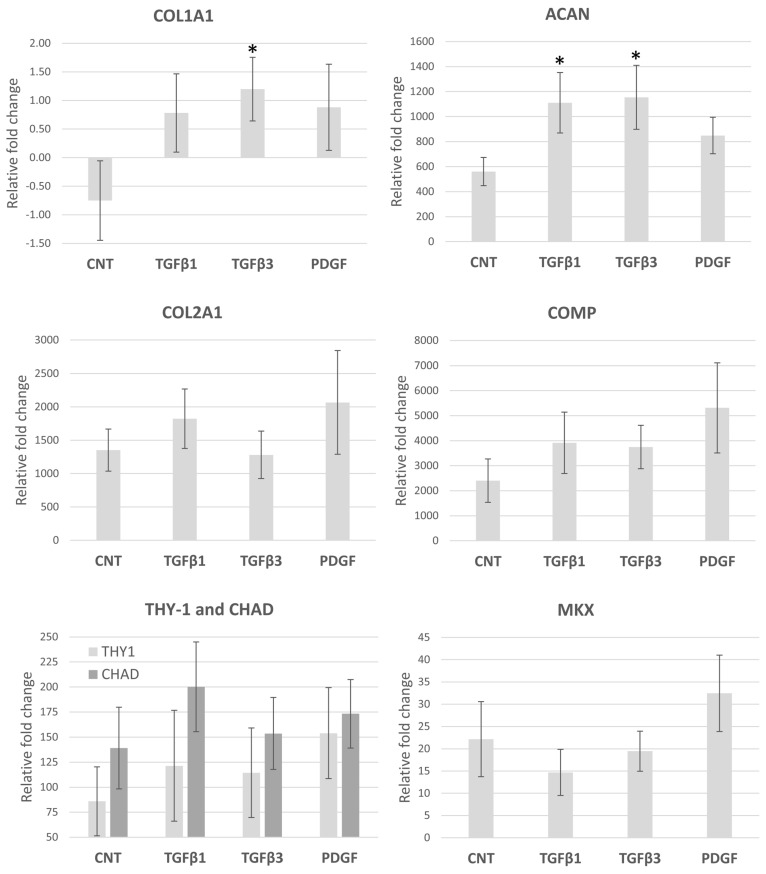
Gene expression profiles of ES-MSCs in pneumatospun collagen scaffolds after implantation into human meniscus defects for 5 weeks. Expression levels are relative to undifferentiated ES-MSCs (N = 8–12). (CNT = control non-GF conjugated; TGFβ1 = TGFβ1-conjugated scaffold; TGFβ3 = TGFβ3-conjugated scaffold; PDGFbb = PDGFbb-conjugated scaffold). (* *p* < 0.03).

Additional ex vivo constructs with implanted scaffolds (N = 8–11) were analyzed for mechanical via pushout tests, and scaffolds that were pushed out were preserved for gene expression analysis. Gene expression of ACAN, COL2A1, COMP, CHAD, MKX, and THY-1 was significantly increased in comparison with the monolayer undifferentiated cells (*p* < 0.05), while COL1A1 expression was not significant. In comparison to control scaffolds, we also observed a general increase in gene expression levels in the growth factor-conjugated scaffolds (Figure 8). ACAN gene expression was significantly increased by TGFβ1 and TGFβ3 treatments (*p* < 0.03), while COL1A1 mRNA levels were only significantly higher in TGFβ3-treated scaffolds (*p* < 0.02). No significant differences between treatments were detected for the expression of COL2A1, COMP, CHAD, THY-1, and MKX. Overall, histological profiling and gene expression analyses of the ex vivo constructs demonstrate neotissue formation within live human OA meniscus explants characterized by combined collagen types I and II deposition and upregulation of meniscus-associated markers such as THY-1 and MKX.

### 3.3. TGFβ Enhances Mechanical Integration of Implanted Scaffolds

**Figure 9:**
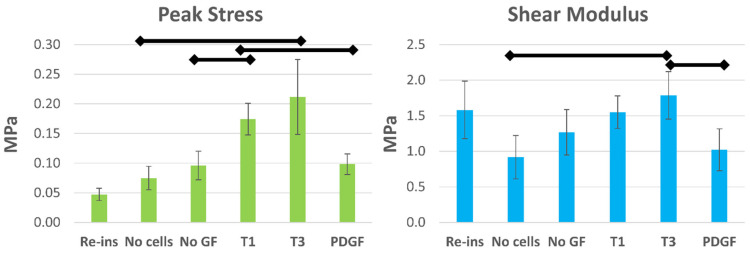
Mechanical pushout testing of pneumatospun collagen scaffolds implanted in ex vivo human meniscus. The peak stress and shear modulus are reported in megapascals (MPa). The horizonal lines indicate significant comparisons (see Section 3.3). Re-ins = re-inserted tissue; No cells = scaffold without cells; No GF = no growth factor; T1 = TGFβ1; T3 = TGFβ3; PDGF = PDGFbb.

Pushout testing was performed to assess the effectiveness of different growth factors on mechanical tissue integration, including: (i) testing of native tissues re-inserted after removal via dermal punch (N = 4) to provide a baseline control; (ii) acellular pneumatospun collagen scaffolds (N = 9); (iii) heparinized and FN-coated scaffolds with cells but without GF (N = 10); and (iv) the three GF-treated scaffolds with cells (N = 8–12). Peak stress and shear modulus were calculated (Figure 9). All conditions resulted in higher peak stress relative to the re-inserted condition (*p* < 0.05). TGFβ1- and TGFβ3-conjugated scaffolds generated significantly higher peak stress values (*p* < 0.004 and *p* < 0.03 respectively) than acellular scaffolds. In cellular scaffolds, TGFβ1 treatment resulted in significantly greater peak stress values (*p* < 0.02), with TGFβ3 treatment approaching significance (*p* < 0.06). Peak stress values for TGFβ1-treated scaffolds were also significantly higher than the PDGFbb-treated scaffolds (*p* < 0.01).

Compared to acellular scaffolds, pushout shear modulus was significantly higher in TGFβ3-treated scaffolds (*p* < 0.04, Figure 9), while that for TGFβ1-treated scaffolds approached significance (*p* < 0.06). Scaffolds treated with TGFβ3 also resulted in a higher shear stress modulus compared to those treated with PDGFbb (*p* < 0.05).

### 3.4. Proof of Concept Laminated Scaffolds

While the pneumatospun scaffolds show promise to repair meniscus tissues, the mechanical properties were low (tensile modulus of ~0.1 MPa) [23]. Previously, we had shown that electrospinning coaxial scaffolds (PLA and collagen) resulted in a higher tensile modulus of ~50 Mpa [15]. However, it is challenging to electrospin thick scaffolds, and the nanofibrous scaffolds are dense, which prevents extensive cellularization. As a proof of concept, we explored combining electrospinning and pneumatospinning to create laminate scaffolds. Our hypothesis was that this combination will permit cellular infiltration, increase scaffold thickness, and enhance overall mechanical properties.

We pneumatospun collagen on both sides of coaxial electrospun PLA + collagen scaffolds to form three-layered constructs (Figure 2). Mechanical testing of dog-bone-shaped specimens from hydrated laminate scaffolds (Figure 2G) resulted in a tensile modulus of 2.4 ± 1.2 MPa, yield stress of 0.3 ± 0.2 MPa, and a peak stress of 0.6 ± 0.3 MPa. Laminate scaffolds were also fibronectin coated and heparinized for cell seeding and implantation into human meniscus defects (Figure 2H and 4). SEM shows the integration of the interface between the highly dense electrospun scaffold and the more porous pneumatospun collagen scaffold (Figure 3).

**Figure 10:**
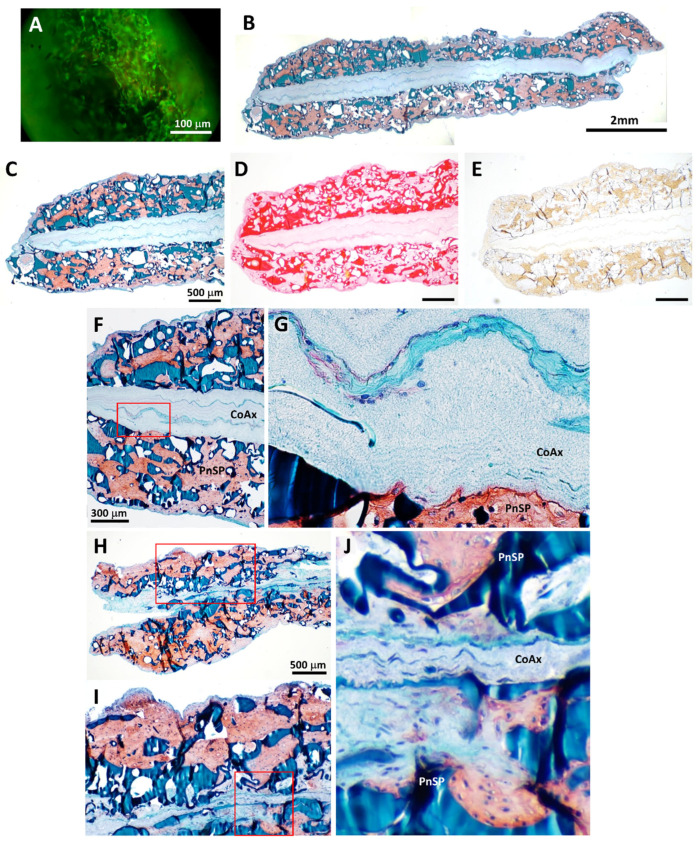
Cell viability and histology in laminate scaffolds after 6 weeks culture. (**A**) Calcein AM fluorescence (green signal) reveals high cell viability and cellular infiltration into the pneumatospun (PnSP) scaffold portion of the laminate scaffold. (**B**–**J**) Histology shows ECM production and cellular distributions. (**B**,**C**) Overview of a laminate scaffold stained with Safranin O (red-orange stain). (**D**) Picrosirius red collagen stain (red stain). (**E**) Collagen type II immunostaining (brown stain). (**F**–**J**) Safranin O stain of interface between pneumatospun and electrospun layers of representative specimens, with insets (**G**–**J**) showing cell infiltration into the electrospun layer.

ES-MSCs seeded into TGFβ3-treated laminate scaffolds showed high cell viability (Figure 10A), uniform cell distribution, and robust neotissue formation in the pneumatospun sections of the scaffold after 6 weeks of culture (Figure 10B). The cells produced ECM that was positive for GAGs (Safranin O), collagen (picrosirius red), and collagen type II (Figure 10C–E). Cellular infiltration into the electrospun scaffold portion of the laminate scaffold was mixed, with some migration of cells into the dense layer (Figure 10F–J).

**Figure 11:**
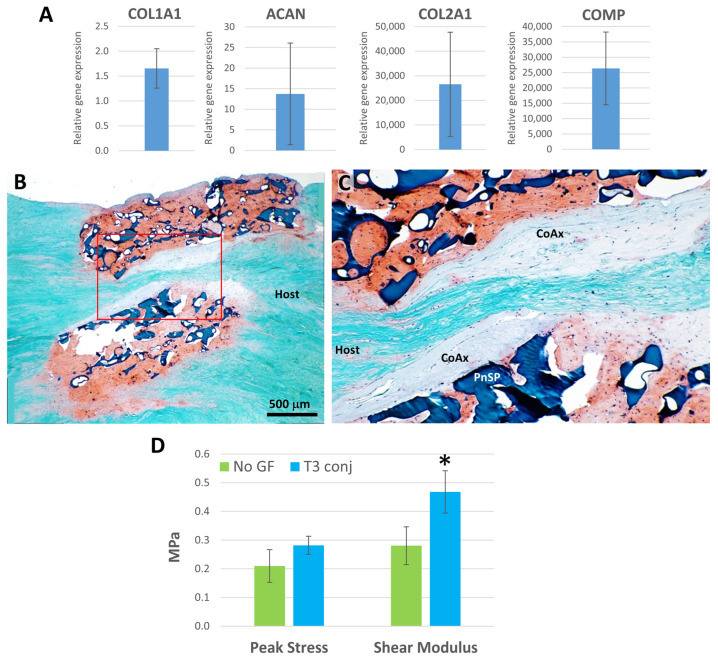
Analysis of laminate scaffolds implanted for 6 weeks into ex vivo human OA meniscus tissue. (**A**) Gene expression profiles of free-cultured laminate scaffolds conjugated with TGFβ3 and seeded with ES-MSCs (relative to undifferentiated ES-MSCs) after 6 weeks in culture (N = 5). (**B**) Laminate scaffold stained with Safranin O (red-orange positive stain). (**C**) In set of panel B showing the pneumatospun (PnSP) layers, the coaxial scaffold (CoAX), and adjacent host meniscal tissue (Host). (**D**) Mechanical pushout testing (peak stress and shear modulus (MPa) of laminate scaffolds seeded with ES-MSCs (* *p* < 0.03). No GF = no growth factors (N = 5); T3 = TGFβ3-conjugated scaffolds (N = 10).

ES-MSC-seeded laminate scaffolds were prepared as 3.5 mm by 2–3 mm thick discs (Figure 2H) for in vitro culture for gene expression profiling or for ex vivo implantation for pushout mechanical testing. In comparison to undifferentiated ES-MSCs, the expression of COL1A1, ACAN, COL2A1, and COMP was significantly enhanced in laminate scaffolds cultured for 6 weeks (*p* < 0.05; Figure 11A). Laminate scaffolds implanted into human menisci for 6 weeks generated neotissues that were well integrated into the host tissue (Figure 11B), with extensive cellular infiltration into the coaxial portion of the scaffold (Figure 11C). On mechanical pushout testing, TGFβ3 treatment (N = 10) significantly increased shear modulus (*p* < 0.03) but not peak shear stress compared to scaffolds without TGFβ3 (N = 5) (Figure 11D).

### 3.5. In Vivo Implantation

**Figure 12:**
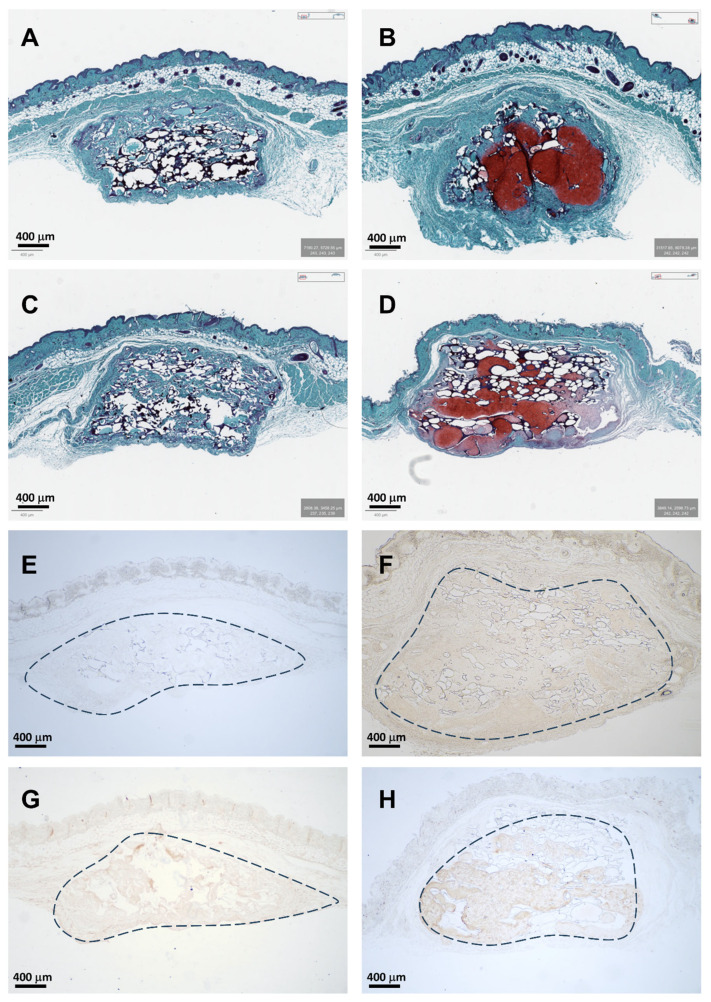
Pneumatospun scaffold and in vivo tissue formation. Representative histology of pneumatospun scaffolds implanted subcutaneously in nude mice for 6 weeks. **Left column** (**A**,**C**,**E**,**G**): Acellular scaffolds (N = 6) remained relatively intact but did not result in substantial neotissue formation. **Right column** (**B**,**D**,**F**,**H**): Scaffolds containing ES-MSCs (N = 6) generated neotissues staining positive for (**B**,**D**) glycosaminoglycans (Safranin O red stain), (**F**) = collagen type I (brown stain), and (**H**) collagen type II (brown stain) (IHC). (Dotted lines indicate implanted scaffold location).

**Figure 13:**
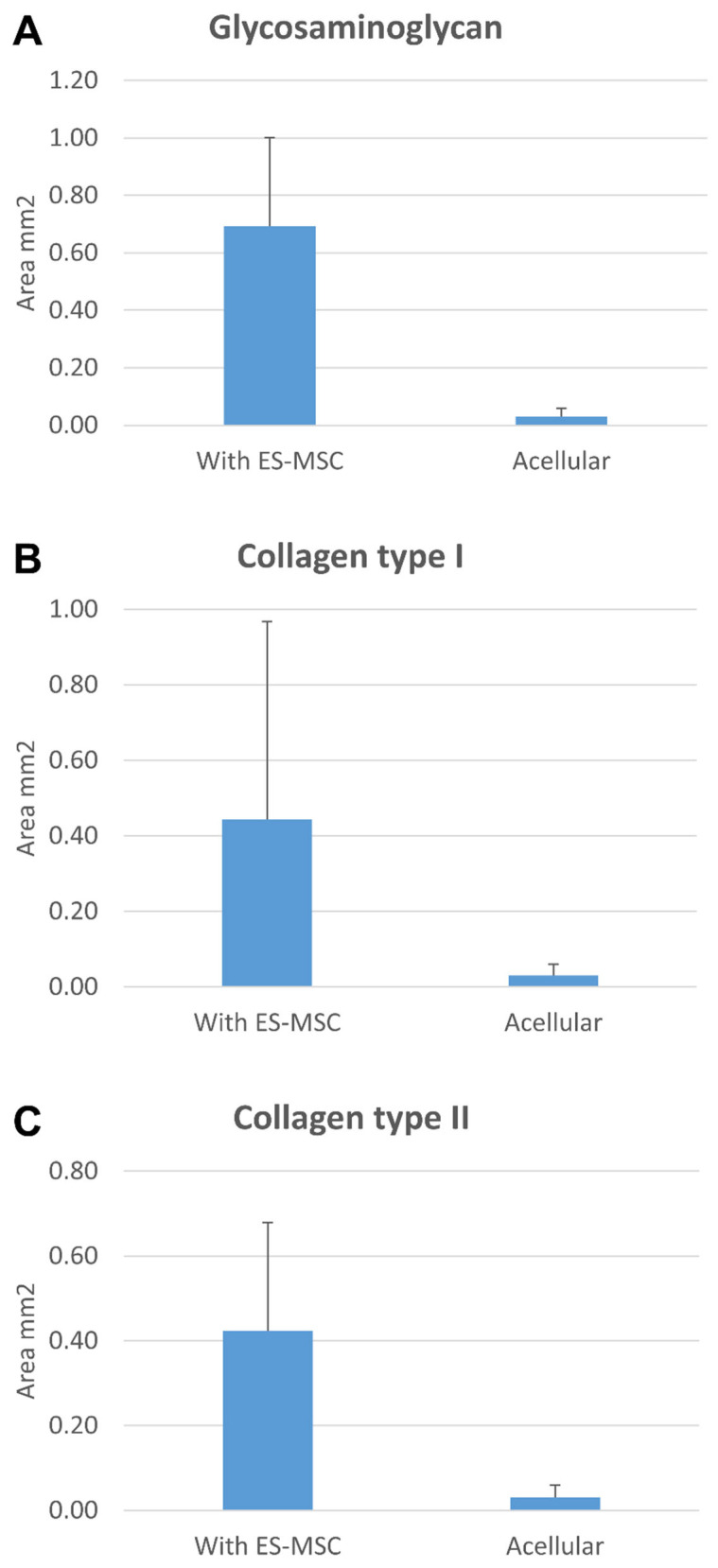
Histomorphometry quantification of IHC stain. ES-MSC seeding (N = 6) significantly increased the area staining positive for (**A**) glycosaminoglycan, (**B**) collagen type I, and (**C**) collagen type II in the neotissue compared to acellular implanted scaffolds (N = 6, *p* < 0.01, paired *t*-test).

Pneumatospun scaffolds were implanted subcutaneously in the dorsum of nude mice with or without ES-MSCs. Scaffolds without cells generated a minimal cellular response at the margins of the implant but very little evidence of neotissue formation (Figure 12 and Figure 13). Scaffold conjugated with TGFβ3 and seeded with ES-MSCs stained positive for glycosaminoglycans, collagen type I, and collagen type II, indicative of fibrocartilaginous formation of meniscus-like tissues.

## 4. Discussion

This study demonstrates that pneumatospun collagen scaffolds, functionalized with heparin for growth factor immobilization and seeded with clinically viable embryonic-derived mesenchymal stem cells (ES-MSCs), support robust neotissue formation and integration in human osteoarthritic (OA) meniscus explant models. We show evidence of cell distribution, tissue formation, and ex vivo meniscal repair by laminating electrospun and pneumatospun layers. Collectively, this work advances regenerative and mechanical strategies for meniscus tissue engineering.

This work builds on earlier studies showing the utility of nanofibrous scaffolds for meniscus repair, where electrospinning has long been a favored technique for fabricating scaffolds capable of mimicking the native extracellular matrix ultrastructure [14,15,17]. Previous investigations report on the challenge of limited cellular infiltration and scaffold thickness posed by dense electrospun networks [18]. We explored pneumatospinning [23] to overcome some of the limitations of electrospinning by rapid and scalable fabrication of millimeter-thick, highly porous collagen scaffolds that exhibit superior cell seeding and distribution, high cell viability, and matrix production. Consistent with other studies of MSCs or meniscus-derived cells [11,12], this work confirms upregulated expression of key meniscogenic markers in ES-MSCs, especially when exposed to immobilized growth factors [15,16,23].

We used heparin conjugation to immobilize TGFβ1, TGFβ3, or PDGFbb, with the TGFβ isoforms, in particular, enhancing both matrix deposition (e.g., increased Safranin O and collagen II staining) and mechanical integration strength. These findings parallel studies reporting on the efficacy of growth factor immobilization in guiding cartilaginous and fibrocartilaginous matrix formation in vitro and in vivo [35,39]. Interestingly, TGFβ1 and TGFβ3 immobilization significantly increased the degree of polarization (DoP) on polarized light imaging of picrosirius red-stained scaffold sections. This marker of enhanced collagen fiber alignment and matrix maturation was associated with improvements in mechanical integration, histomorphometric outcomes, and upregulated meniscogenic gene expression. Mechanical integration, assessed by pushout shear stress and modulus, reached levels comparable to or exceeding earlier reports of ex vivo engineered neotissues evaluated by pushout testing [40,41,42,43,44,45,46] and was consistent with prior studies demonstrating robust neotissue formation and integration in meniscus explant models [15,23,39,41], providing complementary evidence that neotissue fiber polarization reflects functional performance. Together, the data supports the strategy of scaffold functionalization and potential for clinical translation of our approach.

Nude mice provide a convenient model to study cellular behavior, scaffold bioresorption, and neotissue formation in vivo [39,52]. We had previously reported that electrospun collagen scaffolds were largely bioresorbed and underwent tissue remodeling by 6 weeks after in vivo subcutaneous implantation [39]. Pneumatospun scaffolds, on the other hand, remained relatively intact. We also found evidence that ES-MSCs in pneumatospun scaffolds generated fibrocartilaginous neotissue indicative of meniscus-like tissue formation by 6 weeks. These are preliminary in vivo results and do not provide evidence of meniscal repair but indicate that the pneumatospun collagen scaffolds support the tissue formation potential of ES-MSCs in vivo.

Advanced in vitro and ex vivo systems have become increasingly important for preclinical evaluation (see the official NIH policy statement (https://www.nih.gov/news-events/news-releases/nih-prioritize-human-based-research-technologies (accessed on 29 April 2025))). We had previously characterized an ex vivo model to reproducibly assess cartilage–scaffold integration and quantify tissue bonding strength [53]. We used a similar ex vivo model for meniscus tissue integration to provide a translationally relevant approach that supports biomechanical assessment of scaffold integration aligned with evolving research policies.

A range of laminate and composite scaffolds have been tested for meniscus regeneration using diverse material combinations and cell sources. Reported configurations include silk fibroin and wool keratin blends [54], meniscus ECM with methacrylated gelatin [55], PLA with natural hydrogels [24], cellulose and chitosan composites [56], polycaprolactone (PCL) with silk fibroin [36], as well as multicomponent scaffolds containing PCL, silk fibroin, gelatin, and ascorbic acid seeded with adipose-derived MSCs [57]. Additionally, bioprinted composite hydrogels with aligned synthetic microfibers in methacrylated gelatin using meniscus fibroblasts or BM-MSCs have demonstrated promising results [58]. Collectively, these works highlight incremental improvements in combining mechanical strength with cell-compatible environments. However, many of these composite constructs rely on either dense fiber meshes that limit cellular infiltration or hydrogel-rich matrices with limited capacity for load bearing.

We and others have laminated electrospun scaffolds to address these limitations [24,59,60]. Fisher et al. showed increased stiffness of layered electrospun PCL scaffolds seeded with bovine MSCs [59,60]. We seeded human avascular meniscus cells in a hydrogel composed of collagen type II, chondroitin sulfate, and hyaluronan on layers of electrospun PLA. While these approaches did meet biomechanical requirements, the composite thickness was only a few hundred micrometers [24]. Building on these prior efforts, the present laminate scaffold advances the field by integrating a mechanically robust coaxial electrospun PLA/collagen layer with bioactive, porous pneumatospun collagen exterior layers. Unlike conventional electrospun scaffolds that offer mechanical integrity at the expense of cell infiltration and unlike purely hydrogel-based or single-fiber composite systems that compromise structural robustness, this laminate design leverages outer pneumatospun layers to facilitate cell seeding, viability, and matrix formation, while the electrospun core significantly increased the overall elastic modulus (from 0.1 to 2.4 MPa). The cell migration and matrix elaboration across internal interfaces further support its clinical relevance [14,15,16]. Taken together, these features position this scaffold at least as effective and, in some respects, superior to published prototypes in enabling both mechanical performance and robust cell-mediated integration, aligning with the paradigm of functional biomimicry for complex tissue engineering [15,16].

Scaffold laminate architectures combine the mechanical robustness of synthetic polymers with the bioactivity and porosity of natural collagen, aiming to mimic the structure and function of native meniscus tissue. Effective bonding between electrospun and pneumatospun layers is important for structural integrity while also supporting cell migration and enhancing ECM deposition. Scanning electron microscopy revealed a well-integrated interface between scaffold layers, supporting cell infiltration and neotissue formation. However, further optimization is needed to enhance reproducible cellular migration into the dense electrospun core. Recent literature highlights methods such as chemical crosslinking, interlayer adhesion modifiers, and gradient fiber alignment to reinforce interfacial strength and facilitate robust long-term durability [54,56,57]. Future studies are needed to evaluate interface mechanics, matrix production, and strain transfer, comparing these laminate constructs against emerging composite designs to enhance both biomechanical performance and cellular remodeling for partial meniscus replacement.

We acknowledge the following weaknesses. First, this study utilized an ex vivo human OA meniscus model. In vivo performance, especially under chronic biomechanical loading and the influence of joint homeostasis, remains to be tested. The time frame for neotissue development and integration may not fully reflect long-term repair dynamics seen in vivo. Our ex vivo model does not replicate the complex tear patterns and the loading conditions observed in clinical meniscus injuries. Future studies will, therefore, need to evaluate this system in more clinically representative tears and under physiological loading. We did not attempt to recapitulate the meniscus zonal organization and vascularity. Finally, our investigation of laminate scaffold integration was preliminary; enhancement of mechanical properties, optimization of adhesion between layers, and their long-term durability require a systematic study.

This work demonstrates that pneumatospun and laminate nanofibrous scaffolds, functionalized with growth factors and seeded with clinically relevant ES-MSCs, provide a promising platform for advancing meniscus tissue engineering. Scaffold functionalization via covalent heparinization and growth factor immobilization significantly improved neotissue formation, matrix quality, and biomechanical integration into meniscus tissue defects. The advancement to laminate scaffolds offers a promising solution to the challenges of meeting mechanical and bioactivity requirements, as reflected in growth factor-mediated increases in Safranin O and collagen II staining, collagen fiber polarization, and upregulation of meniscogenic genes, with potential application to partial meniscus replacement grafts. Future studies should include in vivo validation in animal models (knee joint environment) and long-term assessment of tissue maturation and mechanical performance. Additional research is warranted to evaluate the recapitulation of meniscal zonal complexity, optimization of laminate bonding, and the development of off-the-shelf allogenic or stem cell-integrated scaffold products for broader clinical use.

## Data Availability

The original contributions presented in this study are included in the article. Further inquiries can be directed to the corresponding author.

## Supplementary Materials

The following supporting information can be downloaded at: https://www.mdpi.com/article/10.3390/bioengineering13030314/s1, Data Figure S1: Pneumatospinning and Electrospinning equipment; Data Figure S2: Polarized light analysis.
