# Supplementary material for: Pneumatospinning and Electrospinning Scaffolds for Meniscus Regeneration Using Human Embryonic-Derived Mesenchymal Stem Cells
Source: Bioengineering (Basel). 2026 Mar 9;13(3):314. doi: 10.3390/bioengineering13030314 (PMC13023445; doi:10.3390/bioengineering13030314)
Supplement: Supplementary file 1 [file bioengineering-13-00314-s001.zip › bioengineering-4081297-supplementary.pdf]

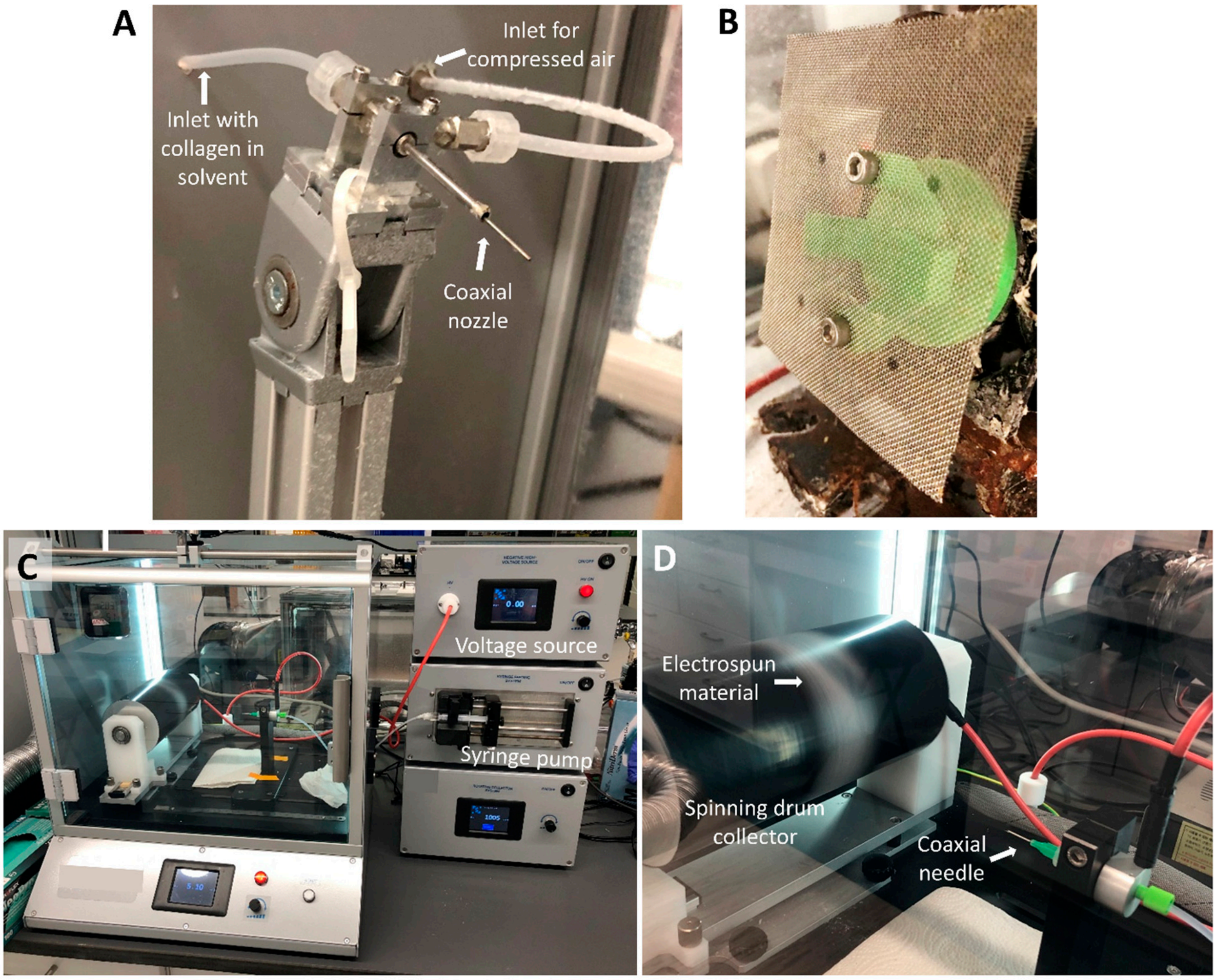

Supplemental Data Figure S1. A. Photograph of a coaxial nozzle showing the inlet lines for collagen solution and compressed air. **B.** Photograph of the detachable mesh collector mounted on a rotating frame during pneumatospinning. **C.** Photograph of electrospinning equipment (Spinbox, Nanoscience) with a voltage source that draws collagen and polylactic acid solutions from the coaxial needle onto the rotating drum. **D.** Photograph of the coaxial needle dispensing nanofibers consisting of a core of polylactic acid within a sheath of collagen, being deposited on the rotating drum collector.

A

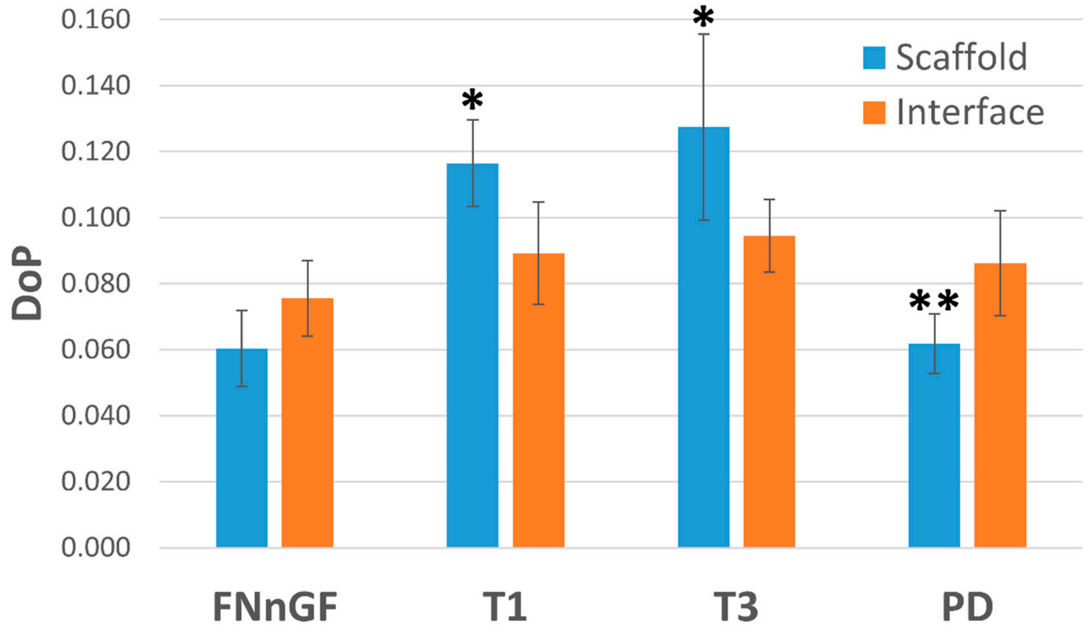

B

Treatment	Scaffold	Interface
No Growth Factor	0.060 ± 0.01	0.076 ± 0.01
TGFβ1	0.116 ± 0.01*	0.089 ± 0.02
TGFβ3	0.116 ± 0.03*	0.095 ± 0.01#
PDGF	0.116 ± 0.01**	0.086 ± 0.01

Supplemental Data Figure S2. Polarized light analysis. The degree of polarization (DoP) was calculated from maximum and minimum intensity ratios for the scaffold alone or at the interface between the scaffold and the native meniscus tissues. **A.** Bar graph showing significantly higher DoP values were observed in TGFβ treated scaffolds compared to no growth factor controls (*P<0.02) and PDGF treatments (**P<0.02). A trend of greater DoP was noted in the interface readings of TGFβ3 treated scaffolds (#P<0.07). **B.** Table showing mean±SEM DoP for each region of interest.
